# Histochemical and Phytochemical Analysis of *Lamium album* subsp. *album* L. Corolla: Essential Oil, Triterpenes, and Iridoids

**DOI:** 10.3390/molecules26144166

**Published:** 2021-07-08

**Authors:** Agata Konarska, Elżbieta Weryszko-Chmielewska, Anna Matysik-Woźniak, Aneta Sulborska, Beata Polak, Marta Dmitruk, Krystyna Piotrowska-Weryszko, Beata Stefańczyk, Robert Rejdak

**Affiliations:** 1 Department of Botany and Plant Physiology, University of Life Sciences, Akademicka 15, 20-950 Lublin, Poland; agata.konarska@up.lublin.pl (A.K.); elaweryszko@wp.pl (E.W.-C.); krystyna.piotrowska@up.lublin.pl (K.P.-W.); 2 Department of General Ophthalmology, Medical University of Lublin, Chmielna 1, 20-079 Lublin, Poland; annawozniak@umlub.pl (A.M.-W.); robert.rejdak@umlub.pl (R.R.); 3 Department of Physical Chemistry, Medical University of Lublin, Chodźki 4A, 20-093 Lublin, Poland; beata.polak@umlub.edu.pl (B.P.); office.stefanczyk@gmail.com (B.S.)

**Keywords:** aucubin, biologically active substances, β-amyrin, β-amyrin acetate, glandular and non-glandular trichomes, oleanolic acid, white dead-nettle

## Abstract

The aim of this study was to conduct a histochemical analysis to localize lipids, terpenes, essential oil, and iridoids in the trichomes of the *L. album* subsp. *album* corolla. Morphometric examinations of individual trichome types were performed. Light and scanning electron microscopy techniques were used to show the micromorphology and localization of lipophilic compounds and iridoids in secretory trichomes with the use of histochemical tests. Additionally, the content of essential oil and its components were determined using gas chromatography-mass spectrometry (GC-MS). Qualitative analyses of triterpenes carried out using high-performance thin-layer chromatography (HPTLC) coupled with densitometric detection, and the iridoid content expressed as aucubin was examined with spectrophotometric techniques. We showed the presence of iridoids and different lipophilic compounds in papillae and glandular and non-glandular trichomes. On average, the flowers of *L. album* subsp. *album* yielded 0.04 mL/kg of essential oil, which was dominated by aldehydes, sesquiterpenes, and alkanes. The extract of the *L. album* subsp. *album* corolla contained 1.5 × 10^−3^ ± 4.3 × 10^−4^ mg/mL of iridoid aucubin and three triterpenes: oleanolic acid, β-amyrin, and β-amyrin acetate. Aucubin and β-amyrin acetate were detected for the first time. We suggest the use of *L. album* subsp. *album* flowers as supplements in human nutrition.

## 1. Introduction

Volatile substances are produced by plants mainly in glandular trichomes, which are spread over aerial vegetative organs [1]. In flowers, volatile organic compounds are emitted from petals during floral maturation [2]. In reproductive organs, essential oils are produced by specialized scent glands, i.e., osmophores, located in various floral elements [3,4]. Plants from the family Lamiaceae are characterized by a high content of essential oils [5]. 

Essential oils are natural multi-component mixtures of products derived from the secondary metabolism in plants. These mixtures and some of their components exhibit a broad spectrum of biological and pharmacological activities. It has been found that the action of whole essential oils is stronger than that of its individual components [6,7,8]. 

Floral volatile organic compounds play an important role in the interaction between plants and various environmental factors. Terpenoids and benzenoids are important components of these substances [9,10,11]. An important function of floral volatile organic compounds is to attract potential pollinators [1,4,12]. The entomophilic *L. album* flowers are characterized by a delicate scent, which is emitted not only by petals [13] but also by the aromatic nectar [14]. The scent of this species of flowers is attractive to many groups of insects that visit these flowers, e.g., bumblebees, honeybees, wild bees, noctuid moths, butterflies, and syrphids [13]. Volatile organic compounds also facilitate communication among plants in the event of parasite attacks. A damaged plant emits a signal to nearby plants, which can then prepare defense responses [15].

Terpenoids exhibit anti-insect, anti-bacterial, and anti-fungal activity. They reduce insect feeding and development and can lead to insect death. Mainly diterpenoids are insect antifeedants [16] (pp. 315–324), while sesquiterpenes may exert deterrent effects against herbivores [17,18]. 

Species from the genus *Lamium* are iridoid-rich plants containing essential oils with pharmacological activity through their anticancer, antioxidant, antibacterial, antiviral, anti-inflammatory, antiarthritic, immunomodulatory, neuroprotective, and wound-healing effects [18,19,20,21]. Differences in the content of volatile compounds in plants can be caused by biotic and abiotic stresses [22,23]. It has been shown that stress most often increases the emission of terpenes in plants, e.g., monoterpenes [24] and sesquiterpenes [25]. It has also been found that the same species may include cultivars that differ in the stress response mechanism associated with terpene production [22].

The composition of *L. album* essential oil may be highly diverse. Various researchers have detected the presence of over 100 compounds therein. The main compounds include 6,10,14-trimethyl-2-pentadecanone, 4-hydroxy-4-methyl-2-pentanone, pentanol [26], tetradecanol-n, benzoate-isopentyl, phytone, undecane–n, neophytadiene, prenol, farnesene-beta-*E*, tridecanol n, dodecanoic acid n, hexadecane-n, dodecanoate-butyl, neophytadiene [27], terpeniol, linalool, spatulenol, bisabolol [28], D-germacrene, β-caryophyllene [18], squalene, octadecanoic acid, tricosane, and pentacosane [29].

Triterpenes are other compounds with broad pharmacological activity contained in *L. album* flowers [18]. They have anticancer, anti-inflammatory, antimicrobial, antitumoral, antiulcerogenic, and antiviral activity [30,31,32,33,34]. Triterpenes detected in *L. album* flowers are represented by oleanolic acid, ursolic acid, and β-amyrin [35,36,37].

Iridoids are one of the main constituents and potential chemotaxonomic markers in representatives of the genus *Lamium* as well. These secondary metabolites are monoterpenes consisting of an iridane skeleton. They mainly occur as glycosides [20,38]. The following glycoside iridoids have been identified in various *Lamium* species to date: lamalbide [39], albosides A and B [40,41], lamalboside and acteoside [42], hemialboside [40], lamiol, caryoptoside, shanzhiside methyl ester, barlerin, 5-deoxylamiol, phytoecdysone, 24-*epi*-pterosterone, penstemoside [29,43,44,45], shanzhiside methyl ester, 6-*O*-syringyl-8-*O*-acetylshanzhiside methyl ester, 6β-hydroxyipolamiide, dehydropenstemoside, sesamoside [46], lamioside [47], and isomers lamiridozin A and B [48]. 

Due to the high content of many secondary metabolites in *L. album* plant organs, extracts from the flowers and shoots are used in the cosmetic industry, especially in hair care formulations [49,50] and as skin-soothing and anti-inflammatory agents [51,52]. Additionally, *Lamium album* can be used as food, e.g., young leaves and stem tips can be boiled and eaten as a vegetable or cooked in soup, or the leaves can be chopped and added to omelettes [53,54]. The leaves can be consumed like spinach [55,56]. Candied flowers of this species are used as edible decorations of desserts [53], whereas fresh ones are used for making wine [57] or decorating salads [56].

Our previous study provided the first analysis of trichomes present in *L. album* flowers. Trichome morphotypes that were characteristic of *L. album* subsp. *album* flowers were distinguished and phenolic acids, flavonoids, and tannins were localized [58]. The petal and stamen indumentum comprised six types of glandular trichomes and two types of non-glandular trichomes. The glandular trichomes present in the corolla were identified as peltate trichomes and short-stalked trichomes with a 1-, 2-, 3- and 4-celled head. The non-glandular trichomes on the corolla were represented by unicellular conical trichomes and 2–5-celled uniseriate unbranched trichomes. On the stamens, which constitute herbal material together with the corolla, long-stalked capitate trichomes and flattened single-celled mechanical trichomes were found [58].

Studies conducted by other authors on *L. album* trichomes focused on leaves, stems, and the calyx. The types of trichomes observed on these organs by other authors largely differed from trichomes located on the corolla in this species [59,60,61].

Plants of the Lamiaceae family are known to produce many biologically active secondary metabolites [18,38,62]. In the scientific literature, histochemical analyses were used to determine the distribution of various compounds in the trichomes of many plant species of this family. However, there was no information about the identification and location of biologically active substances in the tissues of *L. album* flowers, which are a valuable medicinal and pharmaceutical raw material. In our previous study, were detected phenolic acids, flavonoids and tannins in the corollas of this species [58]. The present study is a continuation and extension of previous research and is now focused on other biologically active compounds. The aim of the present study was (i) to conduct morphometric studies of trichomes present on the bilabiate corolla and stamens in *L. album* subsp. *album*, (ii) to determine the location of essential oil and iridoids in corolla epidermis cells and different types of trichomes based on histochemical assays, (iii) to perform quantitative and qualitative analysis of essential oil contained in the corolla, and (iv) to conduct qualitative analysis of triterpenes and quantitative analysis of iridoids contained in the corolla. 

## 2. Results

### 2.1. Morphometric Traits of Trichomes

#### 2.1.1. Corolla

**Figure 1:**
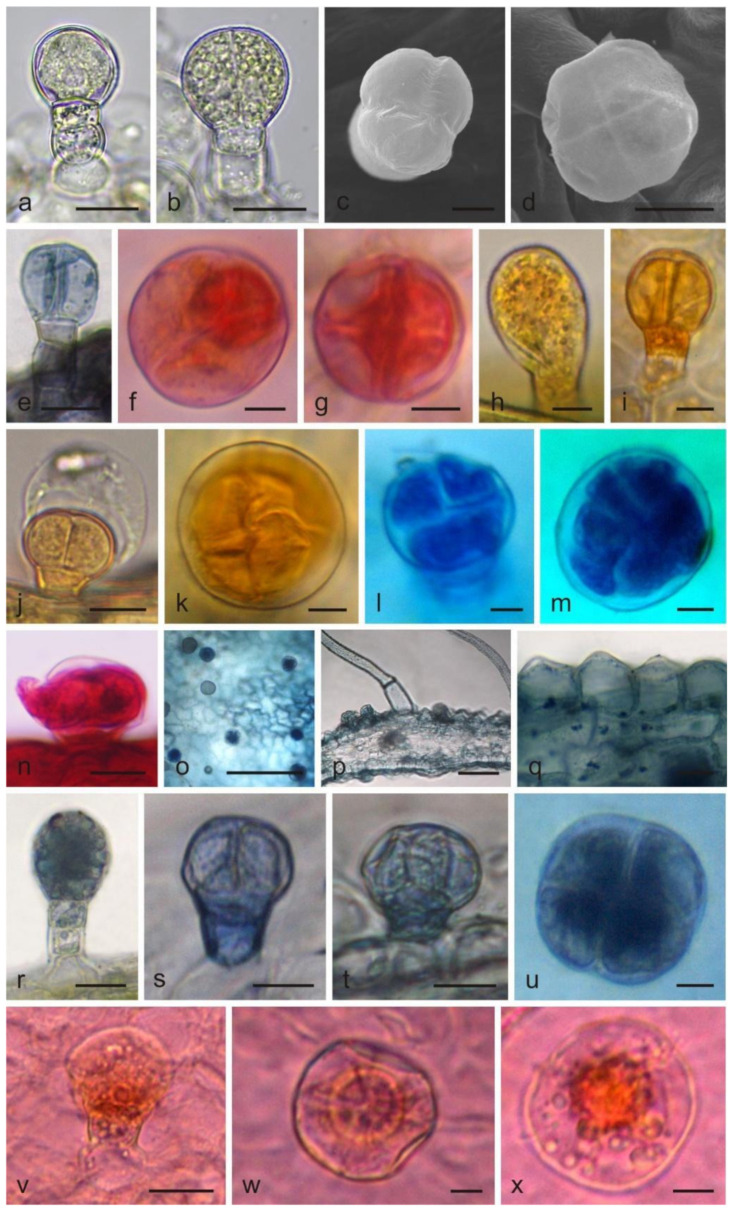
Capitate and peltate trichomes and papillae on the *L. album* subsp. *album* corolla and the results of histochemical assays. (**a**,**b**) Trichomes without staining (LM); (**c**,**d**) trichomes visible in SEM; (**e**–**x**) trichomes after histochemical assays (LM); (**a**) Capitate trichome with a 1-celled head; (**b**) Capitate trichome with 2-celled head; (**c**) Capitate trichome with 3-celled head; (**d**) Peltate trichome with a 4-celled head; (**e**) Capitate trichome stained blue after Sudan Black B treatment (lipids); (**f**,**g**) Peltate trichomes stained red after Sudan Red B application (total lipids); (**h**–**j**) Capitate trichomes and (**k**) peltate trichome stained orange after Sudan III treatment (lipids); (**l**,**m**) Peltate trichomes stained blue after Nile blue application (acid lipids); (**n**) Peltate trichome stained red after Neutral Red treatment (terpenes/essential oil); (**o**–**u**) Trichomes and papillae stained blue after Nadi reagent application (terpenes); (**v**–**x**) Trichomes stained purple-pink after Godin reagent treatments (iridoids). Scale bars: 500 μm (**o**), 50 μm (**p**), 20 μm (**a**,**b**,**f**,**g**,**j**–**n**,**q**–**x**), 10 μm (**c**,**d**,**h**,**i**).

Glandular (capitate and peltate) trichomes and non-glandular trichomes were observed on the corolla. All capitate glandular trichomes on the corolla were short-stalked and had a 1–4 celled head (Figure 1a–c,e). They differed in the number of head-forming cells and sizes. The capitate trichomes with a 1-celled head had a height of ca. 25.5 μm and a 35.6-μm diameter. The capitate trichomes with a 2- and 3-celled head had a similar size, i.e., ca. a height of 27.9 μm and a 43-μm diameter. The capitate trichomes with a 4-celled head were ca. 29.5 μm in height and their diameter was 39.7 μm (Table 1). The peltate trichomes were composed of one basal cell, a unicellular stalk, and a 4–8-celled head, (Figure 1d,f,g). The trichomes were 29.5 µm high and had a diameter of ca. 80.8 μm (Table 1). 

**Figure 2:**
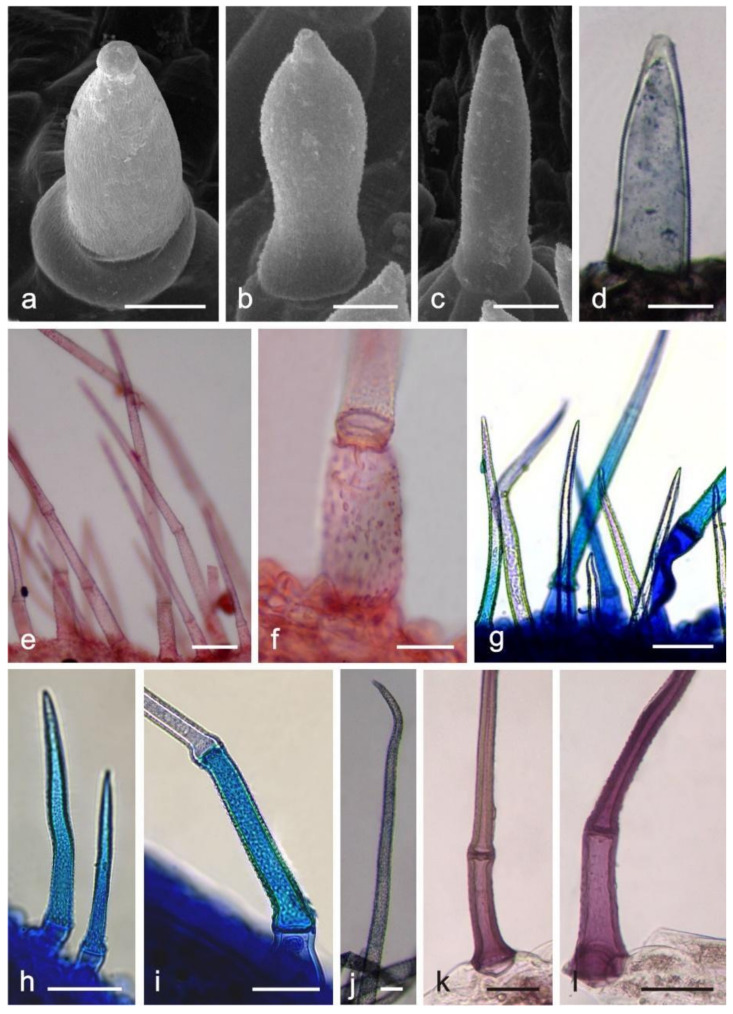
Non-glandular trichomes on the *L. album* subsp. *album* corolla and the results of histochemical assays. (**a**–**d**) Conical trichomes; (**a**–**c**) SEM images; (**d**) LM image; (**e**–**l**) Subulate trichomes; (**e**,**f**) Trichomes stained red after Neutral Red treatment (terpenoids/essential oil); (**g**–**i**) Trichomes stained blue after Nile Blue application (lipids); (**j**) Trichome stained dark blue after Nadi reagent treatment (terpenes/essential oil); (**k**,**l**) Trichomes stained pink-purple after Godin reagent treatment (iridoids). Scale bars: 500 μm (**g**), 100 μm (**e**), 50 μm (**b**–**d**,**h**,**i**,**k**,**l**), 30 μm (**a**), 20 μm (**f**), 10 μm (**j**).

The non-glandular trichomes were represented by conical trichomes (Figure 2a–d), long 1–4 celled subulate trichomes (Figure 2e–l), and 1-celled flattened trichomes. The height of the conical trichomes ranged from 40.9 to 197.6 µm and their diameter was in the range from 21.5 to 72.8 μm, like the long trichomes, whose diameter was similar but the height varied substantially. The long 2–4-celled trichomes were on average almost 5-fold higher than the 1-celled trichomes, and their height ranged from 409 µm to 1273.8 µm. The flattened 1-celled trichomes had an average length of 437.8 µm and a diameter of 35.8 µm (Table 1). 

#### 2.1.2. Stamen

**Figure 3:**
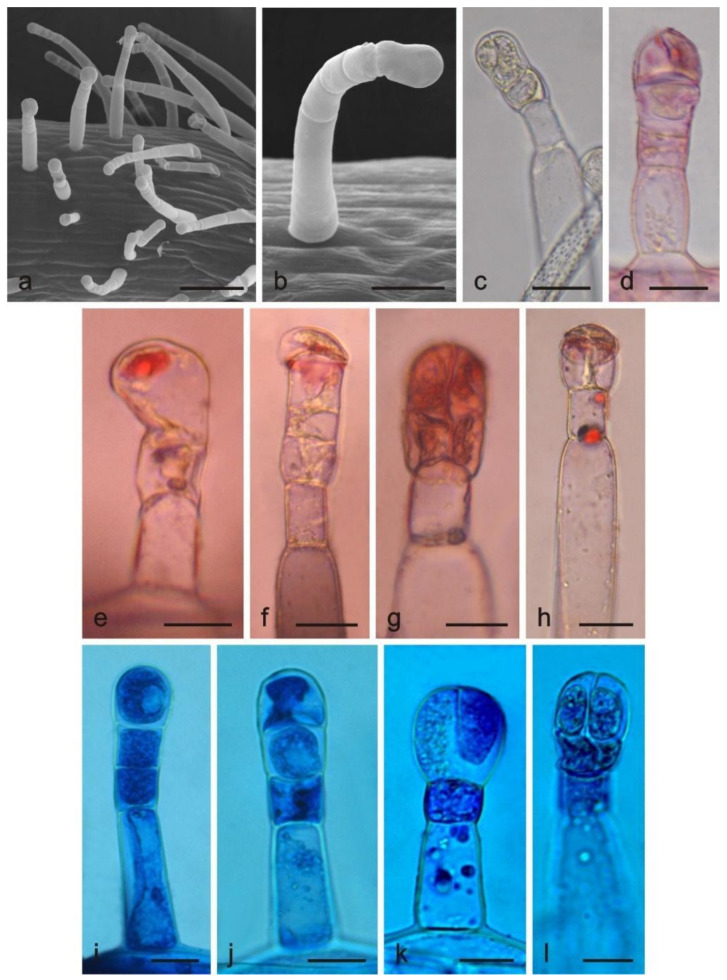
Long-stalked glandular trichomes present on *L. album* subsp. *album* stamens. (**a**,**b**) SEM images of trichomes; (**c**–**l**) LM images; (**c**) Trichome without staining; (**d**–**h**) Trichomes with red-stained content after Neutral Red treatment (terpenoids/essential oil); (**i**–**l**) Trichomes stained blue after Nile Blue treatment (acid lipids). Scale bars: 100 μm (**a**), 30 μm (**b**), 5 μm (**c**–**l**).

Long-stalked glandular trichomes with a 3–5-celled stalk and a 1–4 celled head were present on the surface of anthers and filaments (Figure 3a–l). The trichomes had a height of 157 μm and a diameter of ca. 21 μm. Additionally, there were unicellular non-glandular trichomes on the anthers with a height of 778.6 μm and a ca. 15-μm diameter (Table 1).

### 2.2. Histochemistry

The histochemical assays revealed the presence of various substances, i.e., total lipids, acidic lipids, neutral lipids, terpenes, essential oil, and iridoids, in glandular and non-glandular trichomes located on the corolla, in glandular trichomes from stamens, and in corolla papillae (Table 2).

In the fresh control samples, the secretory product was dark yellow in the short capitate and peltate trichomes on the corolla (Figure 1b), light yellow in the long capitate trichomes on the stamen (Figure 3c), and light yellow in the papillae (not shown). The lipids present in the trichomes stained dark blue after application of Sudan Black B (Figure 1e). After the Sudan Red B treatment, there were red-stained total lipids in the heads of glandular trichomes (Figure 1f,g) and in the papillae. In turn, Sudan III stained the trichome cell content orange (Figure 1h–k). The Nile Blue treatment showed the presence of intense, blue-stained acid lipids in all trichome types (Figure 1l,m, Figure 2g–i and Figure 3i–l). Terpenes/essential oils were present in the papillae and in all types of glandular trichomes, which was confirmed by the treatment of the material with Neutral Red, which stained these substances red (Figure 1n and Figure 3d–h), and with the violet-blue staining Nadi reagent (Figure 1o–u and Figure 2d–j). 

The presence of iridoids in the examined corolla structures was possible to detect with the use of Godin reagent, which stained these compounds pink-purple in the short capitate and peltate trichomes (Figure 1v–x) and purple in the epidermal cells, papillae, and non-glandular trichomes (Figure 2k,l). 

### 2.3. Phytochemical Analyses of the L. album subsp. album Corolla

#### 2.3.1. Analysis of the Essential Oil Composition 

**Figure 4:**
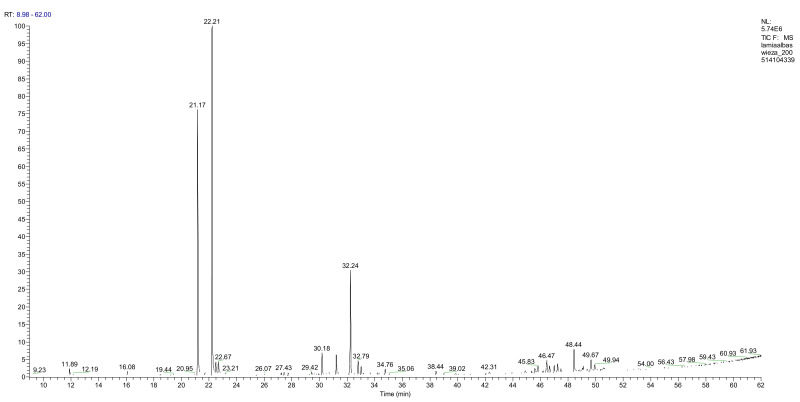
GC-MS chromatogram of *Lamium album* subsp. *album* essential oil obtained from fresh corollas.

**Figure 5:**
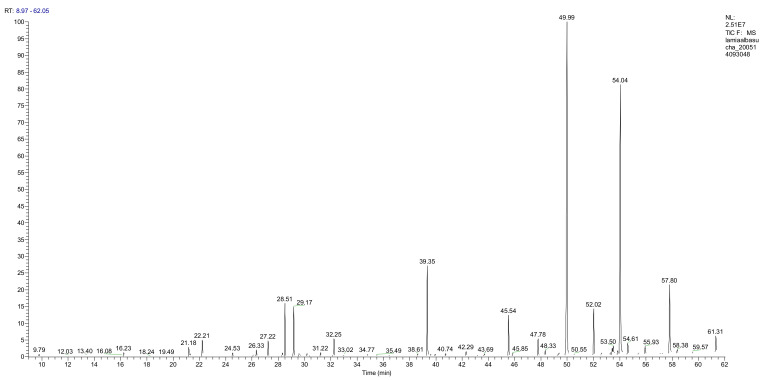
GC-MS Chromatogram of *Lamium album* subsp. *album* essential oil obtained from dry corollas.

The fresh and dried flowers of *L. album* subsp. *album* yielded 0.044 mL/kg and 0.036 mL/kg of essential oil, respectively. The essential oils detected in the fresh and dried flowers were found to contain 33 and 30 components, which constituted 91.1% and 85.6% of the overall composition of the oil, respectively (Table 3 and Table 4). The composition of the essential oil from the fresh flowers was dominated by aldehydes (59.9%) and sesquiterpenes (16.4%), whereas the oil extracted from the dried flowers contained 2.5-fold higher content of sesquiterpenes (42.1%) and alkanes (22%) and over 4.5-fold lower levels of aldehydes (12.9%). Lower contents of alcohols (4.8%), sesquiterpenoids (2.2%), and monoterpenes (1.9%) were determined in the fresh flower oil. Similarly, small amounts of monoterpenes (3.4%), alcohols (2.8%), and sesquiterpenoids (1.3%) were identified in the oil from the dried flowers. Geranial (36.4%) and neral (23.2%) aldehydes, as well as caryophyllene oxide (12.5%) from the class of sesquiterpenes, constituted the highest proportion in the essential oil from the fresh flowers. Branched-chain alkane (21.9%), germacrene D (21.2%), caryophyllene oxide (8.2%), and *E*-β-caryophyllene (6.6%) sesquiterpenes, as well as geranial aldehyde (8.4%), were the dominant compounds in the oil from the dried flowers. Monoterpenoids, alkybenzenes, ketones, and epoxides were present in both oils in the lowest amounts (<1%) (Table 3). The identification of the essential oil compounds from *L. album* subsp. *album* corolla in the chromatograms is presented in Figure 4 and Figure 5.

#### 2.3.2. High-Performance Thin Layer Chromatography (HPTLC) Analysis of Triterpenes

**Figure 6:**
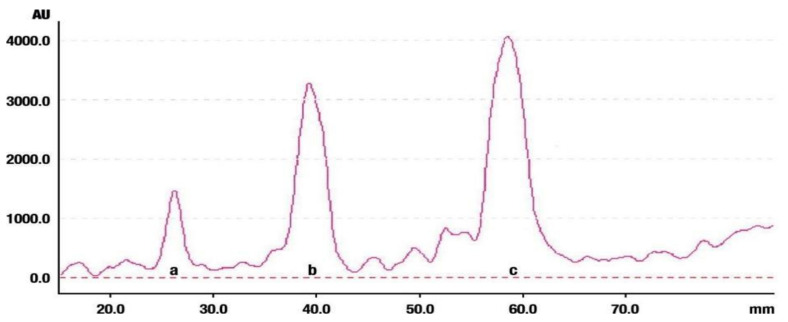
Densitogram of the triterpene standard mixture (a—oleanolic acid, b—β-amyrin, c—β-amyrin acetate). Stationary phase silica gel 60 HPTLC plates, mobile phase n-hexane:dichloromethane:methanol:water (4:5:0.6:0.1 *v*/*v*). Densitometric detection; derivatization with anise aldehyde.

The densitogram of separated triterpene standards is presented in Figure 6. β-amyrin acetate migrates over the longest distance, a moderate distance is covered by β-amyrin, and oleanolic acid reaches the shortest distance. This order of the spot is in accordance with the physicochemical properties of these triterpenes. β-amyrin acetate is the most non-polar compound, whereas oleanolic acid is the most polar among the investigated products. The intensity of oleanolic acid absorbance is lower in comparison to the other triterpenes.

**Figure 7:**
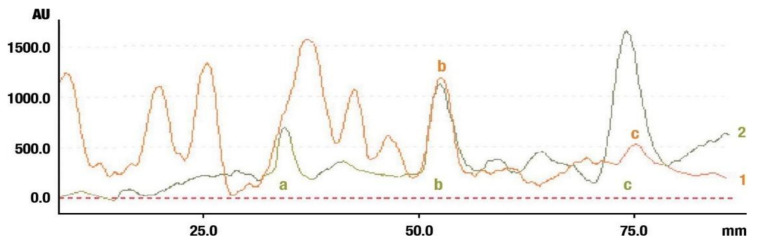
Identification of triterpenes in the chromatogram of the methanolic extract from *Lamium album* subsp. *album* flowers (red, 1) by comparison with the chromatogram of standards (oleanolic acid (a), β-amyrin (b), and β-amyrin acetate (c) (grey line 2). Stationary phase silica gel 60 HPTLC plates, mobile phase n-hexane:dichloromethane:methanol:water (5:4:0.6:0.1 *v*/*v*). Densitometric detection after derivatization with anise aldehyde.

**Figure 8:**
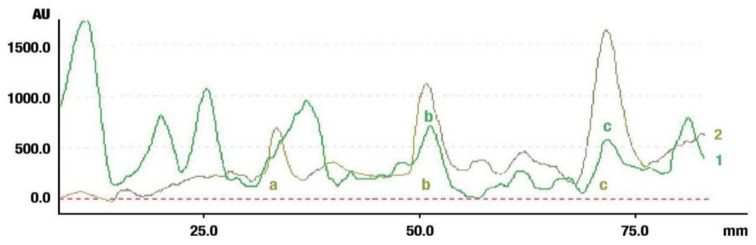
Identification of triterpenes in the chromatogram of the ethyl acetate extract from *Lamium album* subsp. *album* flowers (green, 1) by comparison with the chromatogram of standards (oleanolic acid (a) β-amyrin (b), and β-amyrin acetate (c) (grey line 2). Stationary phase silica gel 60 HPTLC plates, mobile phase n-hexane:dichloromethane:methanol:water (5:4:0.6:0.1 *v*/*v*). Densitometric detection after derivatization with anise aldehyde.

**Figure 9:**
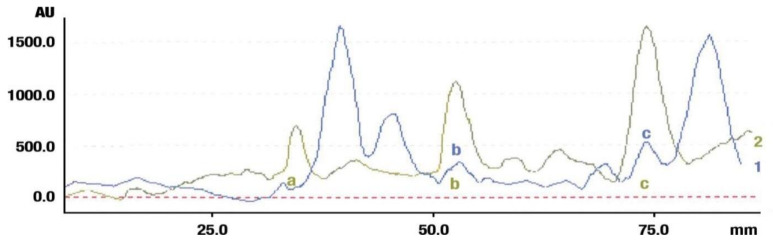
Identification of triterpenes in the chromatogram of the heptane extract from *Lamium album* subsp. *album* flowers (blue, 1) by comparison with the chromatogram of standards (oleanolic acid (a) β-amyrin (b), and β-amyrin acetate (c) (grey line 2). Stationary phase silica gel 60 HPTLC plates, mobile phase n-hexane:dichloromethane:methanol:water (5:4:0.6:0.1 *v*/*v*). Densitometric detection after derivatization with anise aldehyde.

The identification of oleanolic acid, β-amyrin, and β-amyrin acetate in various extracts was performed by comparison of the migration distances of standards and the migration distances of extract zones after chromatographic development. A mixture of n-hexane:dichloromethane:methanol:water (5:4:0.6:0.1 *v*/*v*) was applied as the mobile phase. After the development, the plate was derivatized with anise aldehyde. Figure 7, Figure 8 and Figure 9 present identification of triterpene zones in various extracts: methanolic, ethyl acetate, and heptane, respectively.

β-amyrin and β-amyrin acetate were determined in all extracts. The content of the former compound in the methanolic extract from *L. album* subsp. *album* flowers (Figure 7) was almost the same as in the standard in terms of the zone area or height. The content of the latter triterpene in the extract was lower in comparison with the standard concentration. The best solvent to extract both compounds (β-amyrin and β-amyrin acetate) was ethyl acetate (Figure 8), whereas the poorest extraction was achieved when heptane was used (Figure 9). The zone of oleanolic acid was hard to detect in all extracts due to co-elution with other compounds. 

To solve this problem, further experiments were undertaken after choosing the wavelength of oleanolic acid. 

**Figure 10:**
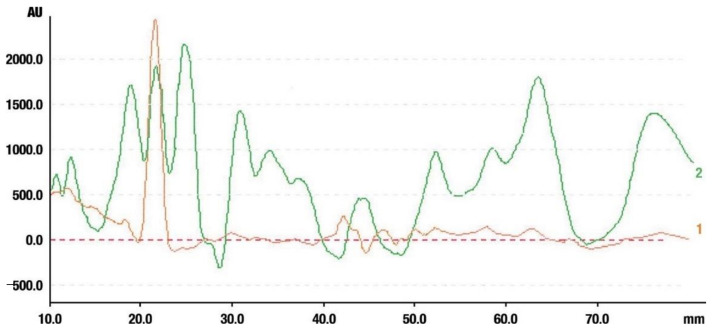
Comparison of the separation of the standard solution of the oleanolic acid standard (red line, 1) and the ethanolic extract from *Lamium album* subsp. *album* (green line, 2). Stationary phase silica gel 60 HPTLC plates, mobile phase n-hexane:dichloromethane:methanol:water (5:6:0.4:0.1 *v*/*v*). Detection after derivatization with 560 nm.

The identification of oleanolic acid in the chromatogram of the ethanolic extract from *L. album* subsp. *album* is presented in Figure 10.

The chromatographic systems developed for triterpenes were used for quantitative analysis of these compounds in plant raw materials. The chemical structures of the isolated triterpenes are shown in Table 5.

#### 2.3.3. Spectrophotometric Analysis of Iridoids—Aucubin

We decided to use the spectrometric method for quantitative analysis of iridoids (aucubin) in the *L. album* subsp. *album* extract. These compounds were expressed as aucubin (Table 5). 

The average content of iridoid aucubin in the *L. album* subsp. *album* flower extract determined with the use of the calibration curve was 0.0015 ± 4.3 × 10^−4^ mg/mL (Table 6).

## 3. Discussion

### 3.1. Trichomes

The histochemical assays used in the present work detected lipids, essential oils, terpenoids, and iridoids in the glandular trichomes of *L. album* subsp. *album* corollas. Since we have not found such information in the literature so far, we present these data for the first time to fill the gap in the knowledge of this issue. Our previous study showed the presence of phenolic compounds: tannins, phenolic acids, and flavonoids in trichomes of this species [58]. The present study is a continuation and extension of our previous research.

The results indicate a heterogenous composition of the secretion in the peltate and capitate trichomes present on *L. album* subsp. *album* corollas. Based on histochemical reactions, the presence of lipids was detected in the cells of all types of glandular trichomes. However, the most intense color reaction was observed in the case of the peltate trichomes (Sudan Red B), which indicates the highest lipid content in the essential oil. The results of the present study largely confirm the findings reported by some authors, who observed the greatest amounts of essential oil contained in peltate trichomes [1,64].

The histochemical assays revealed a less intense reaction to the presence of lipophilic substances in the capitate trichomes of the *L. album* subsp. *album* corollas than in the peltate trichomes. Similarly, other studies reported that capitate trichomes in species from the family Lamiaceae produced fewer lipophilic compounds [65,66].

The presence of terpenes/essential oil was detected with the histochemical method (Nadi reagent) in all glandular trichomes of the *L. album* subsp. *album* corolla. Trichomes of many other species of this family have also been found to produce terpene-rich essential oils [67,68,69]. Terpenes present in essential oil are probably responsible for the pharmacological activity/medicinal properties and protective function in plants from the family Lamiaceae [68].

Previous studies suggest that different types of trichomes serve specific functions [70,71]. This hypothesis is confirmed by the results of the investigation on trichomes in *Hyssopus officinalis* subsp. *aristatus* (Lamiaceae) conducted by Venditti et al. [72]. The authors showed that short-stalked capitate trichomes produced polysaccharides, only terpenes were present in medium-stalked capitate trichomes, and a complex mixture of phenolic and terpenoid compounds was contained in peltate trichomes. Diversity in the substances produced in different types of glandular trichomes was also observed in *Stachys annua* subs. *annua* [73]. In this species, the presence of lipids, terpenes, and polyphenols was detected in peltate trichomes, lipids and terpenes were mainly produced by short-stalked capitate trichomes, and lipids exclusively were contained in long-stalked capitate trichomes.

In plants from other families, different types of trichomes may specialize in the production of specific secretion. In *Lippia scaberrima* (Verbenaceae), terpenoids were determined as the major components contained in large peltate trichomes and phenolic compounds dominated in small capitate trichomes [74]. In turn, Sacchetti et al. [75] showed that diterpenes were produced in secretory trichomes with a 2-celled head in *Calceolaria adscendens* (Scrophullariaceae), and secretory trichomes with an 8-celled head in *C. volckmanni* produced triterpenes. The dependence of the chemical composition of glandular trichome secretion on the morphology of the secretory head has been suggested in investigations of *Cordia verbenacea* (Boraginaceae) conducted by Vantrella and Marinho [76]. The authors have found that the secretion of globular trichomes consists mainly of essential oil, whereas the secretion of reniform trichomes is dominated by flavonoids.

As shown by our previous observations, short-stalked capitate trichomes with a 2-celled head in *L. album* flowers function non-synchronously [58]. The capitate trichomes located next to each other in *L. album* subsp. *album* were in different stages of metabolic/secretory activity: some stained intense blue in the reaction with Toluidine Blue O (phenolic compounds), other trichomes stained green-blue, and some did not stain at all, which suggested that they were in the post-secretory phase or degenerated. Similar results in a study of capitate trichomes with a 2-celled head present on *Euphrasia stricta* (Orobanchaceae) leaves were reported by Haratym and Weryszko-Chmielewska [77]. This phenomenon seems to be one of the causes of the differences in the amounts of various substances in different types of trichomes.

Mechanical protection is most often reported as the function of non-glandular trichomes, which are referred to as tector or clothing trichomes [78,79]. However, the histochemical assays used in the present study revealed the presence of lipids, terpenes, and iridoids in the cells of the non-glandular trichomes in the *L. album* corollas. In a previous study, we also detected tannins, flavonoids, and pectins in this type of trichomes [58]. Bioactive compounds were also found in non-glandular trichomes of several other Lamiaceae and Verbenaceae species [80]. Similar results were reported in studies of non-glandular trichomes from *Dracocephalum moldavica* leaves, which contained various types of metabolites: phenolic compounds, flavonoids, lipids, and terpenes [69]. These data indicate that non-glandular trichomes not only play a passive protective role but also can actively influence the biotic elements of the environment.

### 3.2. Essential Oil

Significantly low amounts of essential oil were extracted from the fresh and dried *L. album* subsp. *album* flowers (0.044 mL/kg and 0.036 mL/kg, respectively). The level of essential oil in fresh flowers was 22% higher than the content in the dried material. Similarly, half the amount of essential oil from dried vs. fresh *L. album* raw material was obtained by Morteza-Semnani et al. [26]. In turn, Flamini et al. [5] demonstrated considerable variability of essential oil yields in the range of 0.01–0.31% in different *Lamium* species. Previous studies demonstrated that the most volatile components of essential oil evaporate during drying, which may reduce the amount of extracted oil [81,82]. Moreover, some components of essential oil can be decomposed through oxidation and hydroperoxidation at elevated temperatures [83]. Drying at increased temperatures may also result in more intense degradation of the cell wall and plasma membrane, which may exert an effect on plasma membrane permeability [84]. In contrast, greater amounts of essential oil from dried flowers were extracted by, e.g., Zheljazkov et al. [85], Mashkani et al. [86], and Silva et al. [87]. 

In the present study, 33 and 30 compounds were identified in the oil extracted from the fresh and dried raw material, respectively. Other investigators reported the presence of a 4-fold higher number (130) [88] or by an over 30% higher number (43) [26] of components in *L. album* essential oil. In turn, Alpieva et al. [29] and Mickiene et al. [27] identified significantly fewer components (12 and 22, respectively).

The composition of the oil extracted from the dried *L. album* subsp. *album* flowers differed considerably from the composition of the oil from the fresh material. Aldehydes, which accounted for almost 60% of all essential oil components, constituted the main group of compounds in the essential oil extracted from the fresh flowers; they were followed by sesquiterpenes in this type of essential oil. The dried flower oil was dominated by sesquiterpenes (42%), alkanes (22%), and aldehydes (12.9%). Many authors reported similar results of the differences in the composition of essential oil, depending on the extraction method used. They suggest that the composition of oil from the same species largely depends on the mode of raw material processing, i.e., drying and distillation methods [81,86,89,90,91]. Moreover, the differences in the qualitative and quantitative composition of essential oils derived from the same taxon can also be related to other factors, i.e., seasonal variations, plant development phases and age, chemotypes, soil, light intensity, and water availability [88,92,93,94,95,96,97]. As reported by Salman et al. [98], changes in the amount and composition of essential oils extracted from various taxa from the family Lamiaceae are associated with genetic and environmental factors, which also determine the genetic expression and thus influence the chemism of oil components.

Branched-chain alkanes (21.95%) dominated in the oil from the dried *L. album* subsp. *album* flowers, while the largest amounts of geranial and neral aldehydes (36.4% and 23.2%, respectively) were determined in the fresh material. Substantial amounts of geranial and neral, which are constituents of citral, have also been detected in essential oil from other representatives of Lamiaceae, e.g., *Dracocephalum moldavica* [69], *Melissa officinalis* [99,100], and *Thymus dacicus* [101]. These aldehydes are considered to have the widest spectrum of antimicrobial [102], ([103] pp. 2973–3008), and antioxidant [104] activities. 

In the group of sesquiterpenes, the essential oil from the dried *L. album* subsp. *album* flowers was dominated by germacrene D (21%). As reported by Yordanova et al. [18], the content of this compound in *L. album* essential oil may vary substantially (from 6.9% to 46.7%). The content of germacrene D detected by various authors in oil extracted from flowers, bracts, and leaves of four other *Lamium* species, i.e., *L. purpureum*, *L. hybridum*, *L.bifidum*, and *L. amplexicaule*, was lower than in the *L. album* subsp. *album* flowers [5]. In turn, Nickavar et al. [105] reported that germacrene D was the main compound in the essential oil from *L. amplexicaule* aerial parts, and its content was similar to that detected in the *L. album* subsp. *album* material in the present study. As indicated by a study conducted by Hu et al. [106], germacrene has antitumoral activity against various types of cancer and may potentially serve as an antineoplastic drug. Moreover, the antioxidant potential of germacrene D has been supported by various studies [107,108]. 

Considerable amounts of caryophyllene oxide sesquiterpene were detected in the oil from the fresh and dried *L. album* subsp. *album* flowers (12.5% and 8.2%, respectively). Large amounts of this compound have also been identified in oil from *L. maculatum* aerial parts [109]. This component exhibits antimicrobial [85], antifungal [110], and insecticidal properties [111,112] and induces apoptosis of neoplastic cells [113]. 

*E*-β-caryophyllene is another sesquiterpene present in the oil extracted in the present study from the dried *L. album* subsp. *album* flowers (6.6%). The presence of β-caryophyllene in the oil from *L. album* flowers was also reported by Kapchina-Toteva et al. [88] and Yordanova et al. [18] as well as Flamini et al. [5] in other *Lamium* species. As shown by Yordanova et al. [18], the content of β-caryophyllene in *L. album* oil may vary from 1.1% to 13%, depending on plant growth conditions. As demonstrated by various researchers, caryophyllene is attributed a number of biological activities such as anti-inflammatory, anticancer, antibacterial, antioxidant, and local anesthetizing properties. It can also serve as a stimulant for the immune system [18,114,115,116].

As reported by Flamini et al. [5] and Jones et al. [117], germacrene D and β-caryophyllene are the most common sesquiterpenes contained in essential oil extracted from various species of *Lamium*, e.g., *L. hybridum*, *L. bifidum*, *L. amplexicaule*, and *L. purpureum*. In general, sesquiterpenes exhibit strong anti-inflammatory and anti-allergic properties [118].

Many investigators have shown the presence of large amounts of other components in essential oil from *L. album* flowers. In a study conducted by Alipieva et al. [29], squalene was the main component of oil extracted from flowers of this species. Morteza-Semnani et al. [26] identified 6,10,14-trimethyl-2-pentadecanone and 4-hydroxy-4-methyl-2-pentanone, whereas Kovalvoya et al. [28] detected terpineol, linalool, spatulenol, and bisabolol.

### 3.3. Triterpenes

Some of the triterpenes identified in the *L. album* flowers in the present study have been described earlier in this species by other authors, e.g., oleanolic acid and β-amyrin [35,36,37]. In turn, the presence of β-amyrin acetate in the flowers of this species was demonstrated in this study for the first time. We also found that ethyl acetate was the best solvent for the extraction of β-amyrin and β-amyrin acetate. In turn, Paduch et al. [37] reported that heptane extracts contained the largest amounts of β-amyrin in comparison with methanol and ethyl acetate extracts.

Using modern techniques of ultrasonic extraction, Wójciak-Kosior et al. [36] determined the optimal conditions for extraction of high concentrations of oleanolic acid. Investigations of the pharmacological activity of oleanolic acid have demonstrated that this compound may be applied in post-*Helicobater pylori* infection treatment [119]. The antimicrobial activity of oleanolic acid and β-amyrin acetate has been shown to be associated with moderate anti-adhesion properties [31]. Some authors presented oleanolic acid as a potential anti-diabetic compound in plant extracts [120]. 

Antibacterial and anti-inflammatory activity of β-amyrin has been reported in various studies [121,122,123,124]. β-amyrin acetate contained in other plant species exhibited cytotoxic and antiproliferative activity [125,126].

### 3.4. Iridoids

The presence of iridoids in the glandular and non-glandular trichomes from the *L. album* subsp. *album* corollas was demonstrated using histochemical assays and quantified as aucubin in phytochemical analyses of the flower extracts. Aucubin in the extract from the *L. album* subsp. *album* corollas were identified for the first time in the present study. Only Adema [47] detected lamioside, i.e., an iridoid classified as an aucubin-like compound, in the leaves of *L. album* and other *Lamium* species, e.g., *L. garganicum*, *L. longiflorum*, *L. amplexicaule*, *L. purpureum*, and *L. maculatum*. Many researchers have described the presence of other iridoids in *L. album* organs, e.g., caryoptoside, lamalbid, and secoiridoid alboside B [127], lamalbid, caryoptoside, shanzhiside methyl ester, and barlerin [44,45], lamiusides A, B, and C and 6″-O-β-D-glucopyranosylmartynoside [50], and lamalbid and shanzhiside methyl ester [128,129]. As reported by these authors, most of these nonvolatile glycoside compounds exhibit numerous therapeutic properties. As shown by Zhang et al. [130], aucubin has anti-inflammatory, antioxidative, and antiapoptotic properties and is able to reduce the degree of liver ischemia–reperfusion injury significantly. In turn, the results reported by Zhou et al. [131] indicate an inhibitory effect of aucubin on the proliferation and differentiation of fibroblasts, thereby providing protection against pulmonary fibrosis. In turn, the impact of the presence of aucubin on the effectiveness of cosmetic macroemulsions has been shown by Dąbrowska and Nowak [132]. It was found to diminish transepidermal water loss, increase skin hydration, and improve the values of surface evaluation of living skin and skin macrorelief parameters.

## 4. Materials and Methods

### 4.1. Plant Material

Corollas of *L. album* subsp. *album* with stamens collected in the initial period of anthesis from plants growing in the Botanical Garden of Maria Curie-Skłodowska University in Lublin, Poland (51°26′20.7′′ N, 22°51′37.8′′ E) were used in the morphological, anatomical, and phytochemical analyses. The anatomical investigations were carried out in May 2019 and 2020, whereas the phytochemical study was carried out in May 2020. The botanical identification was made by comparison with authentic samples deposited in the Department of Botany and Plant Physiology, University of Life Sciences in Lublin and, additionally, by taxonomy specialist Professor Bożena Denisow, who confirmed the correctness of the identification of the taxon. Moreover, botanical identification of plants was performed using “Illustrierte Flora von Mittel Europa” [133] and “A taxonomic revision of *Lamium* (Lamiaceae)” [134]. Preliminary observations of the morphology and distribution of glandular and non-glandular trichomes were performed with the use of a stereoscopic microscope STM 800 (Microlab).

### 4.2. Light Microscopy—Handmade Preparations

**Figure 11:**
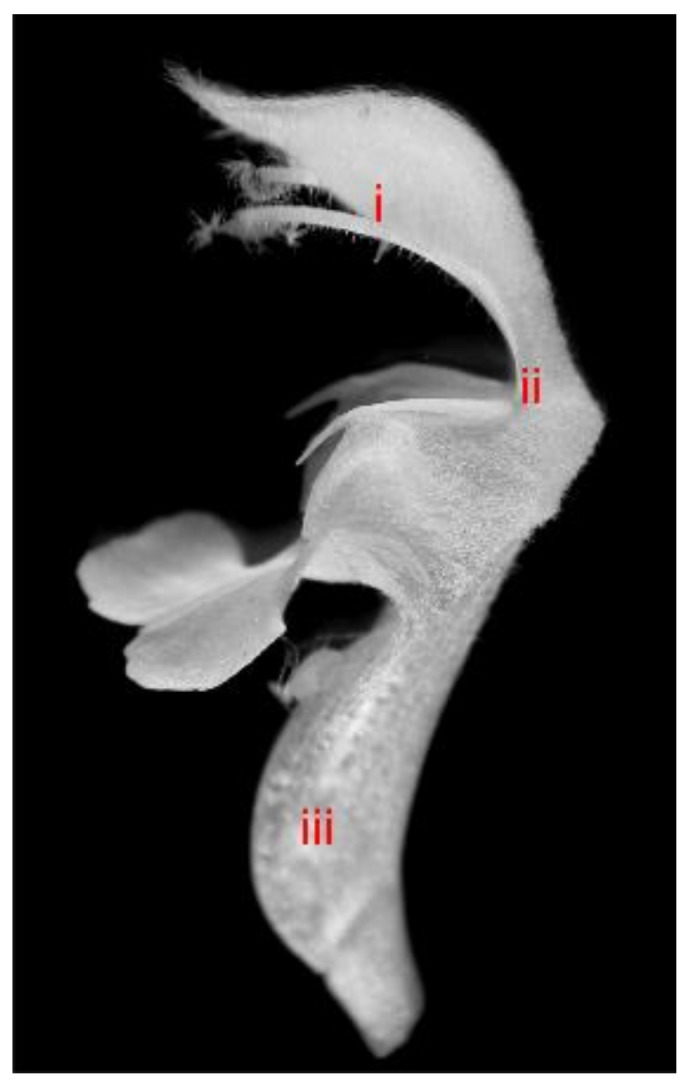
Corolla of *L. album* subsp. *album* with visible sites of section (i–iii).

Corollas and stamens were collected from fresh material of 50 randomly chosen specimens. Using razor blades, handmade cross-sections were made in three areas on the corolla: (i) the edge of the middle part of the upper lip, (ii) the lower part of the upper lip (near the site of fusion with the lower lip), and (iii) the widest point of the corolla tube throat (Figure 11). The plant sections were de-aerated by embedding in a water-glycerine mixture in a 1:1 ratio for 24 h; next, glycerine preparations were made. The stamens were transferred directly on a glass slide and sealed in glycerin (POCH, Gliwice, Poland). The slides were viewed under a light microscope (Nikon, Tokyo, Japan). All types of trichomes present on the abaxial and adaxial corolla surfaces and on the stamens were measured. The height (n = 30) and diameter (n = 30) at their widest point were measured. Photographic documentation was prepared using a Coolpix 4500 (Nikon, Tokyo, Japan) camera coupled to an Eclipse 400 (Nikon) light microscope.

### 4.3. Scanning Electron Microscopy (SEM) Preparations

Stamens and fragments of corollas of *L. album* subsp. *album* collected from the same sites as described above were fixed in a 4% solution of glutaraldehyde in 0.1 M phosphate buffer (pH 7.0) for 12 h at 4 °C. Next, the plant samples were rinsed with the same buffer four times at 20-min intervals and subsequently fixed in 1% osmium tetroxide (*w*/*v*) (Johnson Matthey, Royston, UK) for 1 h and dehydrated in an ethanol series (30, 50, 70, 90, 95% (*v*/*v*) and immersed in anhydrous ethanol (POCH, Gliwice, Poland) twice for 30 min). Then, the plant material was critical-point dried in liquid CO_2_ using a K850 (Emitech, Laughton, East Sussex, UK) Critical Point Dryer. The next stage consisted of spraying the samples with gold (thickness of the gold layer of 20 nm) using a sputter coater K550X (Emitech, Ashford, UK). The material was viewed under a Tescan Vega II LMU (Tescan, Brno, Czech Republic) scanning electron microscope at an accelerating voltage of 30 kV [135]. Microphotographs were taken.

### 4.4. Histochemical Analyses

Fresh free-hand sections of corollas containing glandular and non-glandular trichomes as well as fragments of stamens were subjected to histochemical assays. The following specific reagents were used for detection of the presence of substances: Sudan III (POCH, Gliwice, Poland) [136], Sudan Red B (POCH, Gliwice, Poland) [137] (pp. 874–928) [138], and Sudan Black B (POCH, Gliwice, Poland) [139] for total lipids, Nile Blue (POCH, Gliwice, Poland) for neutral and acidic lipids [140,141], Nadi reagent (POCH, Gliwice, Poland) for terpenes and essential oils [142], Neutral Red (POCH, Gliwice, Poland) for terpenoids (essential oils) [143,144], and Godin reagent (vanillin—sulfuric acid) (POCH, Gliwice, Poland) for iridoids [145]. Six trials/replicates were performed for each reagent. Fresh unfixed and unstained sections of corollas and fragments of stamens were used as a negative control. Photographic documentation was made using a Coolpix 4500 (Nikon) camera coupled to an Eclipse 400 (Nikon) light microscope.

### 4.5. Phytochemical Analyses

#### 4.5.1. Essential Oil Isolation

The essential oils were obtained from 115 g of fresh and 103 g of dried *L. album* subsp. *album* corollas. The drying of the plant material was conducted in a forced air convection oven for two days at 34 °C. The essential oil of dead-nettle was extracted by hydrodistillation method [146]. Both fresh and dried corollas were distilled in 400 mL of water for 3 h in Deryng-type apparatus. The volume of the essential oil was measured; next, the oil was dried over anhydrous sodium sulfate and stored at 4 °C until gas chromatographic determination of its composition. 

#### 4.5.2. Gas Chromatography-Mass Spectrometry (GC-MS)

The chemical composition of the essential oil was analyzed using the GC-MS instrument TRACE GC ULTRA with a POLARIS mass spectrometer Q (Thermo-Finnigan, San Jose, CA, USA). An Equity-5 fused-silica capillary column (30 m × 0.25 mm, 0.25 μm df) (Supelco, Bellefonte, PA, USA) was used. Helium (grade 5.0) was used as a carrier gas. A split-splitless injector was operated in the split mode 1:20 for all chromatographic runs. The temperature of the injector as well as the transfer line temperature was 280 °C. The following temperature programs were applied: 1 min at 35 °C and then a linear temperature increase up to 280 °C at the rate of 4 °C/min. The mass spectrometer was operated in the EI mode at 70 eV; the manifold temperature was 220 °C. The mass spectra were measured in the range 35–400 amu.

Qualitative analysis was carried out via MS spectra, these were compared with the spectra library by means of the NIST MS Search Program (NIST 2.0, 2001), Wiley, and own library, as well as with data available in the literature [63,147,148,149]. Identity of the compounds was confirmed by their retention indices, whereas the retention indices were determined in relation to a homologous series of *n-*alkanes (C_7_–C_30_) under the same opaerationg conditons. 

#### 4.5.3. Ethanolic Extract Preparation for Triterpene Analysis 

The ethanolic extract was prepared according to the Polish Pharmacopoeia V monography on natural products [150]. The flowers of *L. album* subsp. *album* were dried in the shade and air conditions. Next, 25 g of finely divided raw material were filtered through a sieve with 0.315-mm pores. An amount of 0.5 g of *L. album* subsp. *album* sample was weighed and transferred onto a stoppered conical glass flask insert; 50 mL of ethanol was poured into the flask. Next, the flask was placed in an ultrasonic bath (Bandelin, Sonorex, Germany) at 15 °C. The ultrasonic extraction was performed with the use of ultrasounds two times for 15 min. Then, the extract was filtered through a sintered glass funnel (Kisker Biotech GMbh&Co, Steinfurt, Germany). The extract was stored in the refrigerator before further investigations. 

#### 4.5.4. Preparation of Organic Extracts for Identification of Triterpenes

Extracts were prepared by heating 20 g of plant material with 300 mL of the solvent (heptane or methanol, or ethyl acetate) for 5 h at 60 °C in reflux conditions. After this time, the extracts were concentrated under reduced pressure at 30 °C. The final volume of the extract was 100 mL.

#### 4.5.5. Chromatographic High-Performance Thin Layer Chromatography (HPTLC) Analysis of Some Triterpenes

The chromatographic analysis was performed on 100 × 100 mm precoated glass plates with a 0.25-mm layer of silica-HPTLC Kieselgel Si 60 F254 (Merck, Darmstadt, Germany). Before use, the plates were washed with methanol and dried for 20 min at room temperature for activation. The triterpene standards (Sigma Chemical Co, St. Louis, MO, USA) were dissolved in methanol (0.01% *w*/*v*). Then, 5-µL samples of the triterpene standards (oleanolic acid, β-amyrin, and β-amyrin acetate) were spotted using an AS 30 automatic applicator (Desaga CD 60, Heidelberg, Germany) under nitrogen at 2.5 atm of pressure. Chromatograms were developed in horizontal DS chambers (DS II chamber, Chromdes, Lublin, Poland) on a distance of 85 mm. All chromatographic eluent compositions were chosen experimentally. The mixture of n-hexane: dichloromethane: methanol: distilled water (5.0:6.0:0.4:0.1 *v*/*v*) turned out to be the best mobile phase for triterpenes. The separated triterpene zones were detected after the derivatization with the mixture of anise aldehyde, glacial acetic acid, methanol, and concentrated sulfuric acid (VI) (0.5:10:85:5 mL). 

After spraying the derivative reagent solution and drying, the plates were heated at 100 °C in the oven for 5–10 min. The triterpene zones were detected at VIS or UV (366 nm) light.

#### 4.5.6. Selection of the Analytical Wavelength for Oleanolic Acid

**Figure 12:**
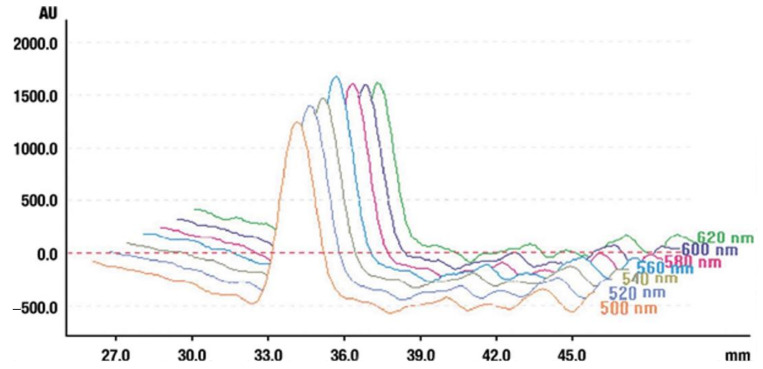
Determination of the analytical wavelength for oleanolic acid. Stationary phase silica gel 60 HPTLC plates, mobile phase n-hexane:dichloromethane:methanol:water (4:5:0.6:0.1 *v*/*v*). Densitometric detection at 560 nm after derivatization with anise aldehyde.

As mentioned earlier, there were problems with the detection of oleanolic acid in the extracts and we had to decide to choose a characteristic wavelength for this compound. Therefore, in the next stage of our experiment, to enhance both the measurement accuracy and detection limit, we applied 0.1 mm of the densitometer slit. The oleanolic acid zone on the chromatographic plate was scanned in the range of 300–680 nm of the light spectrum. The range wavelength of 500–620 nm was promising. Figure 12 shows the selection of the analytical wavelength for oleanolic acid—560 nm. This wavelength was chosen for further investigations. 

#### 4.5.7. Spectrophotometric—Quantitative Analysis of Iridoids

Quantitative analysis of aucubin was performed according to Polish Pharmacopoeia VI [146] and literature data [151]. 

**Figure 13:**
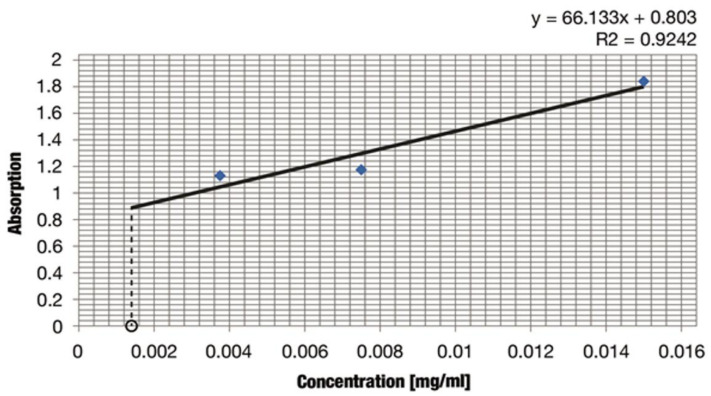
Determination of aucubin content in mg/mL in the ethanolic extract from dried *Lamium album* subsp. *album* flowers.

Aucubin quantitative analysis was performed with the use of the spectrophotometric method. To build the calibration curve, aucubin stock solutions were prepared with the following concentrations: 0.015 mg/mL, 0.0075 mg/mL, and 0.00375 mg/mL). An amount of 0.5 mL from each solution was transferred to three test tubes. Then, 1 mL of methanol, 2 mL of a 4-dimethylaminebenzaldehyde solution containing 0.5 mol/L of hydrochloric acid (Ehrlich reagent), and 1.5 mL of distilled water were added to the test tube. The mixture was heated for 3 min in a boiling water bath (EkoTerm TW 20, Julabo, Schwalbach, Germany). Next, the test tube contents were transferred to a 10-mL flask and diluted to the volume with distilled water. After 15 min, the absorbance of the blue solutions was measured using a Genesys 20 spectrophotometer (Thermo Fisher Scientific, Spectronic, Texas City, TX, USA) at 590 nm as the analytical wavelength. The experiments were triplicated. The average results were applied to prepare the calibration curve (Figure 13).

## 5. Conclusions

To sum up, we conducted a complete pharmagnostical analysis of the corolla in *L. album* subsp. *album* to determine the distribution, content, and composition of essential oils as well as phytochemical analyses of triterpenes and iridoids. We have shown that these active substances are mainly located in corolla epidermis cells and various types of thrichomes. The content of essential oils in fresh and dried flowers is similar and relatively low. Three triterpenoids were identified in the flowers, i.e., oleanolic acid, β-amyrin, and β-amyrin acetate. The presence of β-amyrin acetate was demonstrated for the first time. Similarly, the presence of iridoid aucubin in the extract from *L. album* subsp. *album* corollas were identified for the first time. *L. album* is a common plant in the flora of Europe and a large part of Asia. It is characterized by abundant and long flowering, which lasts from April to September in Poland; hence, its flowers are a valuable and easily available raw material with nutritional, antioxidant, and anti-allergic effects. Flowers containing various active compounds, e.g., lipophilic compounds and iridoids, are not only used in phytotherapy and cosmetology but can also be recommended as potential supplements in human nutrition and tea additives or food decorations. The consumption of a variety of natural compounds is consistent with the principles of a healthy lifestyle.

## Figures and Tables

**Table 1:** Dimensions of trichomes on *Lamium album* subsp. *album* corolla and stamens.

Types of Trichomes	Height (μm)			Diameter (μm) *	
	Min–Max		Mean	Min–Max	Mean
Corolla					
Short-stalked glandular trichomes					
Peltate		26.0–31.2	29.5 ± 1.8	71.3–97.6	80.8 ± 10.5
Capitate with 1-celled head		23.4–29.0	25.5 ± 2.0	28.6–40.6	35.4 ± 5.3
Capitate with 2- and 3-celled head		23.4–31.7	27.9 ± 2.8	28.6–52.8	43.0 ± 8.6
Capitate with 4-celled head		26.4–31.7	29.5 ± 1.8	34.3–44.9	39.7 ± 0.0
Non-glandular trichomes					
Long 1-celled		102.3–204.6	161.6 ± 34.5	10.2–20.5	15.1 ± 3.6
Long 2–4 celled		409.0–1273.8	753.2 ± 330.8	12.3–25.6	17.9 ± 5.0
Conical		40.9–197.6	125.2 ± 55.8	21.5–72.8	41.7 ± 18.9
Flattened long 1-celled		409.2–480.8	437.8 ± 28.5	25.6–51.2	35.8 ± 9.2
Stamen					
Long-stalked glandular trichomes					
Capitate with 1–4 celled head		15.8–28.6	20.9 ± 4.2	97.6–213.0	157.1 ± 39.5
Non-glandular trichomes					
Long 1 celled		613.8–945.6	778.6 ± 110.5	10.2–18.5	14.6 ± 2.8

Values are means ± SD. * The diameter was measured at the widest place of trichome.

**Table 2:** Histochemical reactions of *Lamium album* subsp. *album* corolla trichomes.

Test	Compound	Colour Observed	Corolla				Stamens
			Papillae	Types of Trichomes			
				Short Capitate	Peltate	Non Glandular	Long Capitate
Sudan Black B	total lipids	dark blue	+	+	+	+	nd
Sudan Red B	total lipids	red	+	+	++	+	++
Sudan III	total lipids	orange	+	++	++	±	+
Nile Blue	acidic lipids	blue	++	++	++	++	++
	neutral lipids	pink	−	−	−	−	−
Nadi reagent	terpenes, essential oil	violet-blue or purple	+	++	++	±	±
Neutral Red	terpenoids, essential oil	red	+	++	++	+	+
Godin reagent	iridoids	violet or pink	±	+	+	++	nd

− negative, ± slightly positive, + positive, ++ strongly positive, nd—not determined.

**Table 3:** Chemical composition of essential oil of *Lamium album* subsp. *album* corollas.

**No.**	**Compound**	**Retention Time** **(min)**	**RRI**	**AI**	**Dry Corollas %**	**Fresh** **Corollas %**
**Monoterpenes**						
Monoterpenes hydrocarbon						
1	α-thujene	9.79	932	924	0.7	-
2	camphene	10.32	946	946	0.1	0.2
3	limonene + β-phellandrene	13.40	1029	-	0.4	-
4	γ-terpinene	14.55	1060	1062	0.2	-
Oxygenated monoterpenes						
1	2,3-dehydro-1,8-cineole	11.89	989	988	-	0.6
2	2-pentyl-furan	12.03	993	984	0.5	0.2
3	terpinen-4-ol	18.95	1178	1174	0.1	-
4	isogeranial	19.23	1186	1160	-	0.2
5	β-citronellol	20.84	1232	1223	-	0.3
6	carvone	21.29	1244	1239	1.4	
7	geranyl acetate	26.07	1386	1379	-	0.4
**Monoterpenoids**						
Oxygenated						
1	α-terpineol	19.44	1192	1186	-	0.4
**Sesquiterpenes**						
Sesquiterpenes hydrocarbon						
1	δ-elemene	24.53	1339	1335	1.4	
2	longicyclene	25.47	1378	1371	-	0.4
3	α-copaene	25.82	1378	1374	0.46	0.1
4	β-elemene	26.33	1394	1398	2.7	0.1
4	(*E)-*β-caryophyllene	27.22	1422	1417	6.6	0.4
5	germacrene D	29.17	1484	1484	21.2	-
6	γ-cadinene	30.19	1517	1513	1.1	2.7
7	δ-cadinene	30.46	1526	1522	0.4	0.2
Oxygenated sesquiterpenes						
1	caryophyllene oxide	32.25	1587	1582	8.2	12.5
**Sesquiterpenoids**						
Sesquiterpenoids hydrocarbon						
1	β-bourbonene	26.10	1387	1387	0.6	-
2	*(E)-*α-bergamotene	27.73	1438	1434	-	0.3
Oxygenated sesquiterpenoids						
1	epi-cubebol	29.59	1497	1493	-	0.2
2	10-epi-cubebol	30.68	1534	1533	0.4	0.6
3	epi-cubenol isomer	33.18	1619	-	0.2	0.3
4	γ-eudesmol	33.65	1635	1630	-	0.3
5	epi-α-cadinol	33.96	1646	1638	-	0.3
6	α-eudesmol	34.20	1655	1652	0.1	0.2
**Alkybenzenes**						
1	p-cymene	13.27	1026	1089	0.1	-
**Aldehydes**						
Aromatic						
1	benzeneacetaldehyde	14.30	1053	-	0.2	-
Aliphatic unsaturated aldehydes						
1	isoneral	18.47	1165	1160	-	0.3
2	neral	21.18	1241	1235	4.3	23.2
3	geranial	22.21	1271	1264	8.4	36.4
**Alcohols**						
Unsaturated aliphatic alcohols						
1	linalool	16.08	1100	1098	0.5	0.7
2	nonen-1-ol	16.23	1104	1152	0.7	-
3	elemol	31.22	1552	1548	1.6	2.2
Cyclic alcohols						
1	borneol	18.47	1165	1165	-	0.2
2	cedrol	32.79	1605	1600	-	1.7
**Alkanes**						
1	n-tetradecane	26.56	1401	1400	0.1	-
2	branched-chain alkane	28.51	1463	-	21.9	-
**Ketones**						
1	piperitone	21.68	1256	1249	-	0.4
2	(*E)*-β-damascenone	26.10	1387	1361	0.6	-
**Epoxides**						
1.	1,2-humulene epoxide	33.02	1613	1608	0.9	0.9
**Unknown**						
1	unknown 1284	22.49	1279	-	-	1.4
2	unknown 1291	22.67	1284	-	-	2.0
3	unknown 1679	34.76	1675	-	-	0.9
					* Total 91.1%	* Total 85.6%

RRI—relative retention index, AI—Arithmetic Index on DB-5 in reference to n-alkanes [63]. * The Table does not include compounds present in the amount <0.1% and unknown compounds <0.5%.

**Table 4:** Chemical structures of the most important compounds isolated from *L. album* subsp. *album* essential oil.

Monoterpenes hydrocarbon								
** 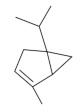 **	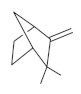		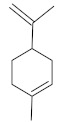	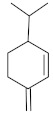	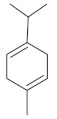			
thujene	camphene		limonene	β-phellandrene	γ-terpinene			
Oxygenated monoterpenes								
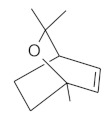	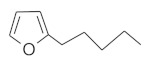		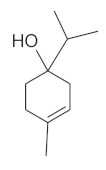	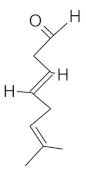	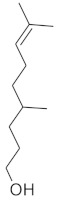	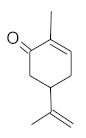	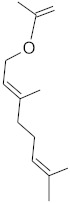	
2,3-dehydro-1,8-cineole	2-pentyl-furan		terpinen-4-ol	isogeranial	β-citronellol	carvone	geranyl acetate	
Monoterpenoids oxygenated								
			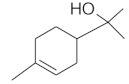					
			α-terpineol					
Sesquiterpenes hydrocarbon								
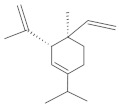	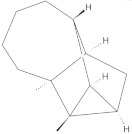		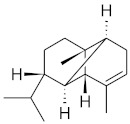	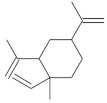	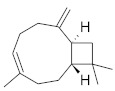			
δ-elemene	longicyclene		α-copaene	β-elemene	(*E)-*β-caryophyllene			
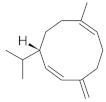	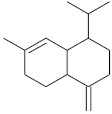		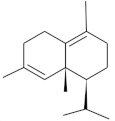					
germacrene D	γ-cadinene		δ-cadinene					
Oxygenated sesquiterpenes				Sesquiterpenoids hydrocarbon				
	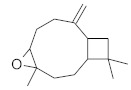				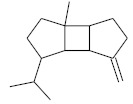	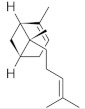		
	caryophyllene oxide				β-bourbonene	*(E)-*α-bergamotene		
Oxygenated sesquiterpenoids								
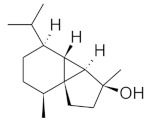	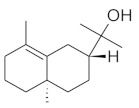		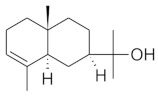	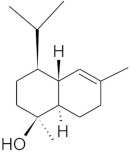				
10-epi-cubebol	γ-eudesmol		α-eudesmol	epi-α-cadinol				
Alkybenzenes	Aromatic aldehydes		Aliphatic unsaturated aldehydes					
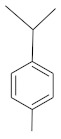		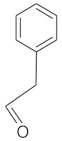	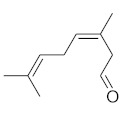	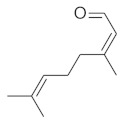		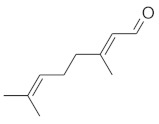		
p-cymene		benzene acetaldehyde	isoneral	neral		geranial		
Unsaturated aliphatic alcohols				Cyclic alcohols				
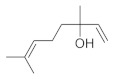		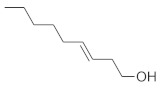		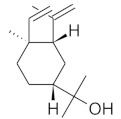	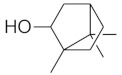	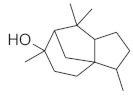		
linalool		3-nonen-1-ol		elemol	borneol	cedrol		
Alkanes			Ketones			Epoxides		
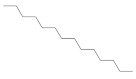			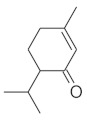	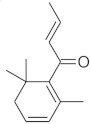		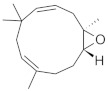		
n-tetradecane			piperitone	(*E)*-β-damascenone		1,2-humulene epoxide		

**Table 5:** Chemical structures of the isolated triterpenes and aucubin.

Chemical Structure			
Triterpenes			Iridoids
β-amyrin	β-amyrin acetate	oleanolic acid	aucubin
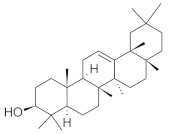	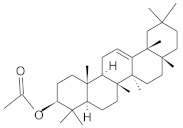	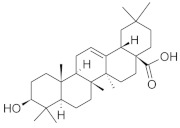	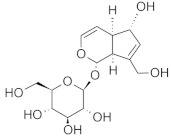

**Table 6:** Average iridoid content in *Lamium album* subsp. *album* flower extract determined as aucubin.

Extract Type	Iridoid Average Content Expressed as Aucubin (mg/mL)	Variance s^2^	Standard Deviation s	s_r_	Confidence Interval 95%
*Lamium album* flowers	0.0015	1.0 × 10^−8^	1.0 × 10^−4^	1.6	0.0015 ± 4.3 × 10^−4^

## Sample Availability

Sample of the compounds are not available from authors.
